# Countering science-related populist speech

**DOI:** 10.3389/fsoc.2023.1275384

**Published:** 2023-11-03

**Authors:** Helle Porsdam

**Affiliations:** Centre for Interdisciplinary Studies of Law, Faculty of Law, SAXO-Institute, University of Copenhagen, Copenhagen, Denmark

**Keywords:** science skepticism, right to science, scientific freedom, scientific dissemination, responsible science

## Introduction

According to the 2021 UNESCO Recommendation on Open Science, the most urgent challenges for the people and the planet are “poverty, health issues, access to education, rising inequalities and disparities of opportunity, increasing science, degradation, climate change, natural and human-made disasters, spiraling conflicts and related humanitarian crises…” (UNESCO, 2021, Preamble).

Many of these global challenges involve science and technology. When we add to this the fact that digital technologies are ubiquitous in our everyday lives, the extent to which we depend on science becomes quite clear. It is no wonder, therefore, that many people are afraid of dual-use science and technology—that is, science and technology that is intended to provide a clear benefit for human beings, but which may be misused, either intentionally or accidentally, to do harm. For some, this fear leads to a distrust of science and scientists, and they feel drawn to misinformation, fake news, and conspiracy beliefs.

Some scholars talk about “science skepticism” (Gerken, 2022); others about “science-related populism”, a variant of populism which does not so much pit *political* elites against the people, but specifically targets *academic* elites (Mede et al., 2021). In this opinion piece, I argue that disinformation and “science-related populism” make it difficult for scientists and scholars to produce the kind of sound research and impartial evidence that decision-makers need to evaluate solutions to urgent global challenges. Scientific freedom and dissemination, and the right to science in general of which they form important parts, offer a promising “tool” for countering such disinformation.

## The right to science

As anti-science populists see it, academics involved in the production of knowledge and truth do not recognize the kind of knowledge that “ordinary” people gather in their lives and experience to be true. Current examples include conspiracist online users promoting “counterknowledge” over “establishment knowledge” (Ylä-Anttila, 2018), and skepticism toward COVID-19 and other vaccines and public health regulations (Bierwiaczonek et al., 2022). The rejection by science-related populists of knowledge produced by academic “elites” typically results in a demand that their own “common sense” must prevail, even regarding decisions about scientists' research agendas, funding, and production of knowledge (Mede and Schäfer, 2020).

As pointed out by the Swiss Commission for UNESCO (2022), lack of trust in science and scientific recommendations is a human rights issue. The situation reflects the current political environment that is divided to the point of being toxic—and where the word “elitist” does not warrant respect but seems instead to have entered the culture wars as a populist signal of criticism against those who think that they may know better. How to avoid that kind of hated “elitism” is the task scientists currently face when they engage with the public and policymakers. Unless they master this engagement, science skeptics may succeed in obstructing much-needed scientific research.

As “a philosophical ideal, a legal promise, a political discourse, and—perhaps—a social movement” (Bishop, 2022, p. xi), the right to science is key to all this. Outlined in both the Universal Declaration of Human Rights (UDHR) and the International Covenant on Economic, Social and Cultural Rights (ICESCR), this human right has been a part of the international human rights system from the very beginning. At its core is a properly trained and adequately supported scientific workforce. If we want to encourage the kind of scientific research that is necessary to solving the serious problems mentioned in 2021 Recommendation, we need to do two things: to focus on scientific freedom under responsibility; and to train scientists to engage better with the public, including science skeptics.

## Responsible scientific freedom

Scientific freedom is accorded a very prominent position in the ICESCR whose Art. 15,3 states that “the States Parties to the present Covenant undertake to respect the freedom indispensable for scientific research and creative activity.” As protected under international human rights law, science must benefit humans and be in harmony with fundamental human rights principles. This means that there can be no scientific freedom, no free pursuit and application of scientific knowledge, without scientific responsibility (Jarvis, 2017).

Whose responsibility are we talking about? Partly that of States Parties to the Covenant, and partly that of scientists themselves. According to Article 15,2 ICESCR positive steps must be taken by States Parties to conserve, develop, and diffuse science (and culture) so that people may realize their right to science (and culture). This implies that States must ensure that research is conducted in a responsible and sustainable manner, that research and technology are promoted, and that scientific culture, public trust, and support for the sciences throughout society in general are strengthened (UNESCO, 2017; Swiss Commission for UNESCO, 2022). States must also promote equal access to research results for all, including across borders, and enable citizen participation in the making of science and science policy [UN Committee on Economic, Social and Cultural Rights (CESCR), 2020].

Citizen participation primarily serves the purpose of soliciting all the knowledge that is out there. We cannot afford to miss out on the good ideas and possible solutions that may come from people outside the scientific world. But citizen scientists and others may also be able to spot dual-use science and technology. The importance of citizen participation in anticipating possible dual-use science and technology comes up in several UN contexts, often in connection with right to science-related themes. In her 2012 report on the right to science, first UN Special Rapporteur for cultural rights Farida Shaheed raises the subject of citizen science as an important element of democratic citizenship, for example (Special Rapporteur in the Field of Cultural Rights, 2012). The rights to science and to culture, mentioned side by side in all four parts of Article 15 ICESCR, both relate to human creativity and the pursuit of knowledge and understanding. They should be considered “in conjunction with, in particular the right of all peoples to self-determination and the right of everyone to take part in the conduct of public affairs” (Special Rapporteur in the Field of Cultural Rights, 2012).

What of scientists themselves? When it comes to scientific integrity, most scientists understand they have a responsibility toward their fellow citizens—but also toward their colleagues within the world of science. Although scientific freedom encompasses the freedom to try out new topics and approaches, the underlying methodology must remain scientifically appropriate. Scientific integrity can be undermined, for example, by forgery and manipulation of data, selection and rejection of unwanted results, manipulation of a representation or an image, and plagiarism and false claims of authorship or co-authorship (Starck, 2006). With regard both to the public and to decision-makers, “the scientific community has responsibilities because it is in a unique position to present information and knowledge that it is developing about the challenges which face humanity and how they might be addressed” (Rhodes and Sulston, 2010, p. 2).

## Scientific dissemination

The open communication of results, hypotheses and opinions lies at the heart of the scientific process and “provides the strongest guarantee of accuracy and objectivity of scientific results” (Rhodes and Sulston, 2010, Preamble). The public cannot exercise their right to science unless scientific knowledge is disseminated so that they understand it. This is acknowledged in Article 15,2 ICESCR. But to whom does the task of communicating scientific results fall? Should scientists themselves be prepared to let the public know the results of their research, or are professional science journalists better equipped to do so?

Most scientists consider responding to journalists a professional duty (Peters, 2013). Entering into dialogue with the public is a challenge, though. Many have watched colleagues getting into trouble when attempting to share their research openly—especially when public venues such as social media are involved where it is easy to twist arguments, willfully misunderstand, and turn against people. Very short attention spans can make it difficult to point out misinterpretations and defend arguments.

As trained communicators, professional science journalists may help bridge the gap between scientists, policy makers, and the public. Yet, when they attempt to avoid jargon use and increase straightforwardness in science communication, accuracy and important details may get lost, just as inaccurate arguments may not be countered directly and on the spot. Likewise, science journalists may not necessarily prioritize—or get across to the public—the excitement entailed in the creation and provision of knowledge, which is the core function of science.

Scientists therefore need to be actively involved in disseminating and communicating to the public what their science is about, what its potential is, and why science is important as a public good. Acquiring the skills that are necessary to grasp the attention of the public and their political representatives allows scientists to show the importance of their research, thereby increasing their chances of securing future funding. But beyond bettering their own professional career prospects, becoming more visible and reaching the wider public also gives scientists a better chance of engaging with all members of the public as fellow democratic citizens and stakeholders.

Communication between the world of science and the public is not a one-way street. The art of arguing involves dialogue; scientific freedom means freedom to test and to argue, but not to force one's results on others. Talking down to the public is what science-related populists expect scientists to do. Communication skills therefore need to be included as a part of the educational curriculum in STEM and other university disciplines (UNESCO, 2017, Article 14). This does not eliminate the need for professional science journalists. To honor the promise of Article 15,2 ICESCR and its importance for the right to science, scientists would still benefit from being coached by professional science journalists who can show them how to engage with the public in a respectful, non-paternalistic/elitist way.

Openness in and communication about science delivers the best defense against dual-use science—and ultimately also against science-related populism. But it is not enough. More regulation is needed, especially regarding social media. Fake news and disinformation are typically countered through criminal law rules on defamation, threats etc., just as researchers whose radical views border on misinformation may be sanctioned by research ethical norms on good scientific practice. At present, this regulation is often inadequate as criminal law rules date from a time when there were no social media. Also, the norms on research ethics are designed to regulate research activities themselves. They do not so much cover statements that are made as part of the popular dissemination of scientific results.

If we want to counter science-related populist speech, we therefore need to update rules regarding regulation of what is said on social media. Importantly, this must be done in such a way that it does not violate freedom of expression and scientific freedom.

## Author contributions

HP: Writing—original draft.
